# Some Suggestions in Regard to the Treatment of Gonorrheal Rheumatism

**Published:** 1880-09

**Authors:** William A. Byrd

**Affiliations:** 407 Jersey Street; Quincy, Illinois


ARTICLE II.

### SOME SUGGESTIONS IN REGARD TO THE TREATMENT OF GONORRHEAL RHEUMATISM.

BY WILLIAM A. BYRD, M. D., ETC., QUINCY, ILLINOIS.

It is not my intention to draw upon any of the authors who have written upon gonorrheal rheumatism, as that would but take up the time of the reader, who doubtless has consulted the "authorities" upon the subject time and again.

I have met with but four cases of the malady in my practice. In three of these cases but one joint was affected, and that was the knee, in the other case one knee and one wrist were the only joints that were affected.

The attack in one case occurred the sixth day after the discharge from the penis was first noticed, and that was the case in which both knee and wrist were involved, In two of the other cases the rheumatism showed itself between the second and third week after the commencement of the discharge. In the fourth case there were three different attacks; the first coming on the second day after I had commenced treating the patient for stricture of the urethra, of large caliber, by dilatation. There had been no active discharge from the urethra for over a year, the patient having consulted me for the relief of a slight but persistent gleet, that I found, by the use of bulbous sound, to be incident to three strictures of large extent. The second attack occurred about four months after the cure of the first, and also the curing of the gleet, and came on a week after a gonorrhea contracted during a debauch. After recovering, another debauch was followed by a gonorrhea, and in eight days the rheumatism manifested itself. The left knee was attacked each time in this case and it was also the suffering joint in two of the other cases, the right knee being the affected joint in the other case.

Recovery was rapid in all but the case that had the two relapses, which was the first case I ever saw. In the treatment of the first attack, I tried splints, anodynes, hot applications, and almost every thing else that I could think would do any good, but the knee went on swelling and the pain becoming intolerable regestering 103° and 104° all the time. Being very young, and what I considered, a very conservative surgeon, I hesitated about using the aspirator, which was just being introduced into practice and had seldom been used in this country, but seeing if something was not done, and that quickly, my patient would die of pain and hyperpyrexia, I aspirated the joint, drawing off six ounces of fluid at the first operation. One hour after the aspiration the pain was gone and the temperature had fallen from 104° to 100°. As the joint would refill the temperature would rise and the pain recur. The relief from the aspiration was so marked that I repeated the operation whenever the swelling and pain seemed to require it, which was sixteen times during the first attack, withdrawing in one day, with two operations, eleven ounces of fluid. In addition to the aspiration and constitutional remedies, I applied Sayre's knee splint, and strapped the joint with heavy adhesive plaster over compressed sponges. The patient made a good recovery with a scarcely perceptible stiffness of the joint.

In the next attacks he had, and also in the remaining cases, I did not wait for so great an amount of effusion into the joint, and pain, before aspirating, and the operation was required but once. As soon as the fluid was removed from the joint, in the latter cases, I applied Martin's elastic bandage to the joint, which is greatly to be preferred to Sayre's splint and strapping with adhesive plaster, and it is to the elastic, constant pressure of it, that I attribute the non-necessity of second aspirations. In fact I believe with the bandage properly applied aspiration may be entirely dispensed with.

The wrist I did not aspirate, but used Martin's bandage alone on it. It made a good recovery requiring about two weeks, whilst the knee that was aspirated and compressed with a Martin's bandage, in the same patient was well in less than a week.

The last patient that I saw with gonorrheal rheumatism, was a young man of twenty, who had represented to his father that he hurt his knee loading goods upon a wagon. When I called upon him I could find no evidence of external violence and there was not that amount of pain and redness that should have followed a traumatism. There was not the sweating nor high color of the urine of true rheumatism, and the temperature was but slightly above 100°. That condition of things caused me to disbelieve his story of traumatism, so I sent his mother out of the room and boldly asked him when he had first noticed the symptoms of the clap that he was laboring under. He at first denied having it, but when I examined his penis and found it discharging he informed me that he had had it for about two weeks I returned for my aspirator, and got Dr. John W. Niles to assist me, I inserted the needle and withdrew four ounces of fluid, applied a Martin's bandage, ordered a weak solution of sulphate of zinc for injection and a cubebs mixture internally. The father, being very close about money matters, informed me that he would let me know how the young man got along and if he was not doing well he would send for me to see him. That was Tuesday in the afternoon; Friday morning the young man walked into my office apparently as well as he ever was He had remained in bed, wearing the bandage, up to that time, and was still wearing it. I ordered him to continue it for some time as a prophylatic.

I have had most excellent results, lately, in cases of gonorrhea that I have seen at the commencement of the discharge, by treating them with injections of flaxseed tea and fluid extract of opium, and mucilaginous drinks as recommended by Professor Louis Baur of St. Louis in the St. Louis Clinical Record, but I have not had sufficient experience to know whether the treatment will answer in all cases or not.

The urine can generally be kept bland and unirritating with the following:

℞. Brom. Pot. - - - - ʒ ii

Acetate Pot. - - - ʒ iv

Camph. Tr. Opium - f ℥ ii

Oil Cubebs - - - - f ʒ iij

Glycerine - - - - f ℥ iij

Syrup qs. to - - - f ℥ xii

Mix S. give a tablespoonful every two to four hours.

Or if that should not agree with the stomach the following may at times be substituted with advantage:

℞. Benzoic Acid - - - ʒ iii

Bi Carb. Soda - - ʒ vi

Sugar - - - - - ℥ iv

Mix S. give a teaspoonful it a tumbler of water every two hours.

Of course quinine, iron, and other tonics must be had recourse to as the necessities of each individual case requires, as also a proper regulation of the diet and bowels.

With the treatment above described, I found my patients got well much more quickly than I had been led from the teachings of the books to expect. 407 Jersey Street, August 14th, 1880.

